# Narrative exposure therapy for survivors of human trafficking: feasibility randomised controlled trial

**DOI:** 10.1192/bjo.2021.1029

**Published:** 2021-10-28

**Authors:** Francesca Brady, Amy Chisholm, Eileen Walsh, Livia Ottisova, Leonardo Bevilacqua, Claire Mason, Martha von Werthern, Teresa Cannon, Christina Curry, Kemi Komolafe, Rachel Elizabeth Robert, Katy Robjant, Cornelius Katona

**Affiliations:** The Helen Bamber Foundation, London, UK; Woodfield Trauma Service, Central and North West London NHS Foundation Trust, London, UK; and Department of Clinical, Educational and Health Psychology, University College London, UK; Freedom from Torture, London, UK; The Helen Bamber Foundation, London, UK; and Traumatic Stress Clinic, Camden and Islington NHS Foundation Trust, London, UK; Traumatic Stress Clinic, Camden and Islington NHS Foundation Trust, London, UK; Department of Clinical Psychology, University of East Anglia, UK; School of Psychology, University of East London, London, UK; Department of Clinical, Educational and Health Psychology, University College London, UK; The Helen Bamber Foundation, London, UK; The Helen Bamber Foundation, London, UK; The Helen Bamber Foundation, London, UK; The Helen Bamber Foundation, London, UK; Freedom from Torture, London, UK; and Vivo International, Konstanz, Germany; The Helen Bamber Foundation, London, UK; and Division of Psychiatry, Faculty of Brain Sciences, University College London, UK

**Keywords:** Post-traumatic stress disorder, individual psychotherapy, randomised controlled trial, trauma, human rights

## Abstract

**Background:**

Human trafficking is a grave human rights violation and a major public health concern. Survivors present with high rates of mental health problems including post-traumatic stress disorder (PTSD). Studies of effective treatments for PTSD in survivors of human trafficking are lacking. Narrative exposure therapy (NET) is an effective PTSD treatment for multiple, prolonged and complex trauma, but its efficacy has not been rigorously tested in survivors of human trafficking.

**Aims:**

To test the feasibility and acceptability of a randomised controlled trial (RCT) offering NET as a treatment for PTSD in trafficking survivors with a history of multiple traumatic events, as well as providing preliminary evidence regarding its efficacy (trial registration: ISRCTN95136302).

**Method:**

A single-blind RCT compared NET with a wait-list control in survivors of trafficking with PTSD (*n* = 25). In the NET arm of the study, participants attended a mean of 17 sessions.

**Results:**

NET was well tolerated by participants. There were significant reductions in PTSD, depression and anxiety symptoms post-treatment in the NET group but no significant change in the wait-list group.

**Conclusions:**

The results indicate that NET is a promising and acceptable treatment for trafficking survivors. Psychological therapy in an RCT design can be safely delivered to this vulnerable group, although modifications are required to ensure their holistic needs are properly addressed.

Human trafficking, the recruitment of people within or across national borders for the purposes of exploitation, is a grave human rights violation and a major public health concern. Although its scale is hard to quantify, as of 2016, an estimated 24.9 million people were trafficked into different types of exploitation globally.^1^ Individuals are exploited for a range of purposes including forced labour, illegal drug cultivation, domestic work and forced sex work. Many survivors of trafficking endure prolonged periods of exposure to hazardous conditions, abuse and violence, captivity and restriction of movement, as well as restricted access to adequate nutrition or healthcare.^2^ Trafficked individuals typically experience multiple or repeated traumatic events, including physical violence, sexual abuse, and threats to them and their loved ones.^3^ Systematic reviews have demonstrated a high prevalence of physical and psychological health problems in survivors of trafficking, including depression, anxiety and post-traumatic stress disorder (PTSD).^3^

In addition to the high prevalence of mental health problems, many trafficking survivors face significant barriers to accessing evidence-based treatments. These include destitution, homelessness and legal stressors, as well as feelings of fear, shame and mistrust of others, which can prevent help-seeking.^4^

In spite of calls for trafficking survivors to be provided with comprehensive and culturally appropriate healthcare, and despite recent advances in the literature around the risk and protective factors for the development of psychopathology in this population, there is a dearth of research into effective treatments.^5,6^ Efficacy and real-world effectiveness studies are needed to develop and validate evidence-based therapies for this extremely vulnerable group.

Narrative exposure therapy (NET) is an evidence-based treatment for PTSD relating to multiple or prolonged traumatic events.^7–9^ Through detailed narration, NET processes and contextualises traumatic memories and helps individuals to establish a coherent autobiographical narrative of their experiences.^10^ NET has been widely used in lower- and higher-income countries and with a range of different client groups, including asylum seekers and refugees.^11,12^ NET is therefore a good candidate for treating PTSD in survivors of human trafficking. A case series with ten female trafficking survivors with a history of sexual exploitation (who received 10–19 NET sessions) found preliminary evidence for its acceptability and effectiveness.^13^ Treatment resulted in significant reductions in PTSD severity and general distress, which were maintained at 3 month follow-up. Although this indicated potential utility, the case study design limited the generalisability of the results, and it was not clear whether survivors of other types of trafficking might benefit from this intervention.

The current study investigates NET as a treatment for PTSD in trafficking survivors using a randomised controlled trial (RCT) design. Given the barriers to treatment that trafficking survivors may encounter, and the limited literature regarding how best to meet their needs, we aimed to explore the feasibility of delivering NET in an RCT in a real-world clinical setting.

## Aims of this study

The primary aim of the study was to explore the feasibility of conducting an RCT investigating the effectiveness of NET in treating PTSD in survivors of trafficking. The study was designed to:

- explore the acceptability and feasibility of delivering NET to this population within an RCT;
- evaluate the process of recruitment and randomisation and the acceptability of including a wait-list control;
- determine the feasibility of delivering psychological therapy in the absence of specific additional provision of practical support.

Our secondary aim was to investigate the effectiveness of NET in reducing PTSD, depression and anxiety symptoms in trafficking survivors.

As a feasibility study, the sample was potentially insufficiently powered; however, the study was designed to examine the feasibility of testing whether participants receiving NET would show a greater reduction in their PTSD symptoms (clinician-assessed and self-reported symptoms) and in any comorbid symptoms of depression and anxiety compared with those in the wait-list group.

## Method

### Trial design

The trial was a single-centre, blinded, two-armed feasibility RCT with parallel groups: NET and wait-list control (trial registration: ISRCTN95136302).

### Participants

Participants were survivors of trafficking (involving various or multiple forms of exploitation) with a diagnosis of PTSD. Treatment was offered by therapists working at a UK-based charity providing specialist care to individuals who have experienced human rights abuses. This includes psychological therapy, as part of a holistic model of integrated care including legal protection, counter-trafficking safeguarding support, housing and welfare advice, and access to educational and social inclusion activities.

**Fig. 1:**
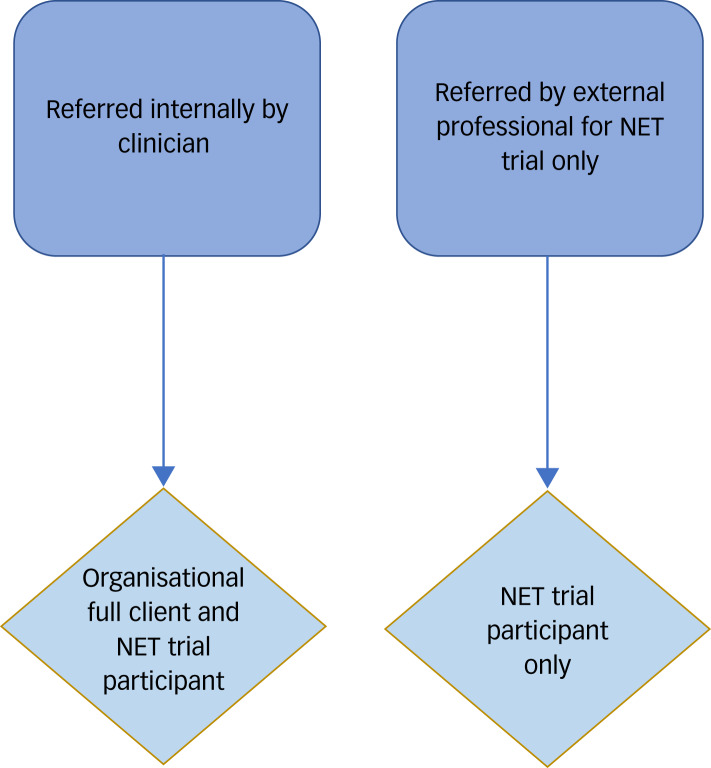
Referral pathway.

Participants were recruited between May 2017 and September 2018 via two pathways (Fig. 1). They were either existing clients of the charity and in receipt of the organisational model of care, or referred by external professionals specifically to receive psychological treatment within the trial (i.e. ‘trial-only’ participants) without access to other aspects of the organisation's support.

All potential participants referred to the study were invited to attend an assessment appointment with a trial therapist. Participants were not offered any incentive for participating in the trial. Travel expenses were reimbursed.

Inclusion criteria were kept as broad as possible: adult survivors of trafficking meeting DSM-5 diagnostic criteria for PTSD;^14^ not having received any type of trauma-focused therapy previously; and willing to engage in NET. Exclusion criteria included: recent suicide attempt or persistent severe self-harm; significant and frequent substance misuse; severe preoccupation with social or legal issues that would interfere with engagement in regular therapy sessions; facing imminent removal from the UK; or currently in a situation of abuse or exploitation.

Fifty-five individuals were assessed for trial suitability during a face-to-face assessment with a clinician; 26 met the study inclusion criteria and gave informed consent to participate. One individual was not progressed in the study owing to instability in their circumstances arising following assessment (Fig. 3). Twenty-five participants were randomly assigned to receive NET immediately (*n* = 15) or to a wait-list control group (*n* = 10). In total, 13 participants completed NET, and seven completed the waiting period (and post-wait assessments). Table 1 shows the sociodemographic variables of the sample at the point of randomisation and the ‘main’ types of trafficking experienced by participants (self-identified). Many had experienced multiple forms of exploitation or other traumatic experiences at various points in their life. All participants completed the Life Events Checklist^15^ as part of their assessment and reported a range of traumatic experiences both within and beyond their experiences of trafficking, including sexual violence, physical assault and witnessing the death of a family member or significant other. However, all participants identified the traumas associated with their period of trafficking as among the primary traumatic experiences that they wanted to address in treatment.

**Table 1:** Demographic characteristics of sample (at baseline) *N* (%) or mean (s.d.)

Variables	NET (*n* = 15)	Wait-list (*n* = 10)
	*N* (%)	*N* (%)
Sex		
Male	4 (26.66)	2 (20.00)
Female	11 (73.3)	8 (80.00)
Interpreter required	5 (33.33)	7 (70.00)
Asylum status		
Refugee status or leave to remain	2 (13.33)	3 (30.00)
Asylum seeker	12 (80.00)	7 (70.00)
Appeal rights exhausted	1 (6.66)	0 (0)
Primary type of exploitation		
Sexual exploitation	8 (53.33)	7 (70.00)
Forced labour	4 (26.66)	2 (20.00)
Domestic servitude	2 (13.33)	1 (10.00)
Other	1 (6.66)	0 (0)
Country of origin		
Nigeria	7 (46.66)	0 (0)
Albania	3 (20.00)	6 (60.00)
India	1 (6.66)	1 (10.00)
Nepal	1 (6.66)	0 (0)
Vietnam	1 (6.66)	0 (0)
China	1 (6.66)	1 (10.00)
South Africa	0 (0)	1 (10.00)
Kenya	0 (0)	1 (10.00)
Philippines	1 (6.66)	0 (0)
Trial-only participants	8 (53.33)	6 (60.00)
Mean age at start of trial	26.73 years (s.d. = 9.35)	32.8 years (s.d. = 10.96)

### Outcome measures

The main clinical outcome in the study was overall PTSD symptom severity, measured through a clinician-administered diagnostic assessment and a self-report scale.

The Clinician-Administered PTSD Scale (CAPS-5)^16^ is the ‘gold-standard’ clinician-administered structured diagnostic interview for PTSD. Items are rated from 0 (absent) to 4 (extreme/incapacitating) with a maximum possible score of 80. Different cut-offs have previously been suggested to indicate clinically significant change on the CAPS-5.^17^ Given our small sample size and the relatively high variability in scores, we deemed it inappropriate to apply a very stringent method of assessing reliable change (two standard deviations from the mean) and instead used Halvorsen's^17^ other suggested method: a change of >30% from baseline score.

The PTSD Checklist for DSM-5 (PCL-5)^18^ is a 20-item self-report measure of PTSD symptom severity. Items are rated from 0 (not at all) to 4 (extremely) based on the frequency or impact of the symptom. A sum score was calculated for PTSD severity (maximum score = 80). Suggested clinical cut-offs for this measure are scores of 31−33.^19^ As participants had experienced multiple traumatic or difficult life experiences, they were reminded to focus on the main traumatic experiences that they had previously identified during completion of their psychological and CAPS-5 assessments when providing their responses to the PCL-5.

#### Secondary clinical outcome measures

The Shutdown Dissociation Scale (Shu-Dis)^20^ is a 13-item measure of the physical signs of dissociation. This measure was included because of its clinical utility in NET, as it indicates the potential likelihood of dissociation, as well as common signs and symptoms that will need to be managed and addressed during therapy. Items are scored from 0 (not at all) to 3 (several times a week/often) depending on the current frequency of symptoms. A sum score was calculated for dissociation severity (maximum score = 39).

The Patient Health Questionnaire (PHQ-9)^21^ and the Generalized Anxiety Disorder Scale (GAD-7)^22^ are, respectively, a nine-item measure of depression and a seven-item measure of anxiety severity. They are both widely used and have been validated. Items are scored from 0 (not at all) to 3 (nearly every day). A sum score was calculated for symptom severity (depression maximum score = 27; anxiety maximum score = 21).

### Procedure

**Fig. 2:**
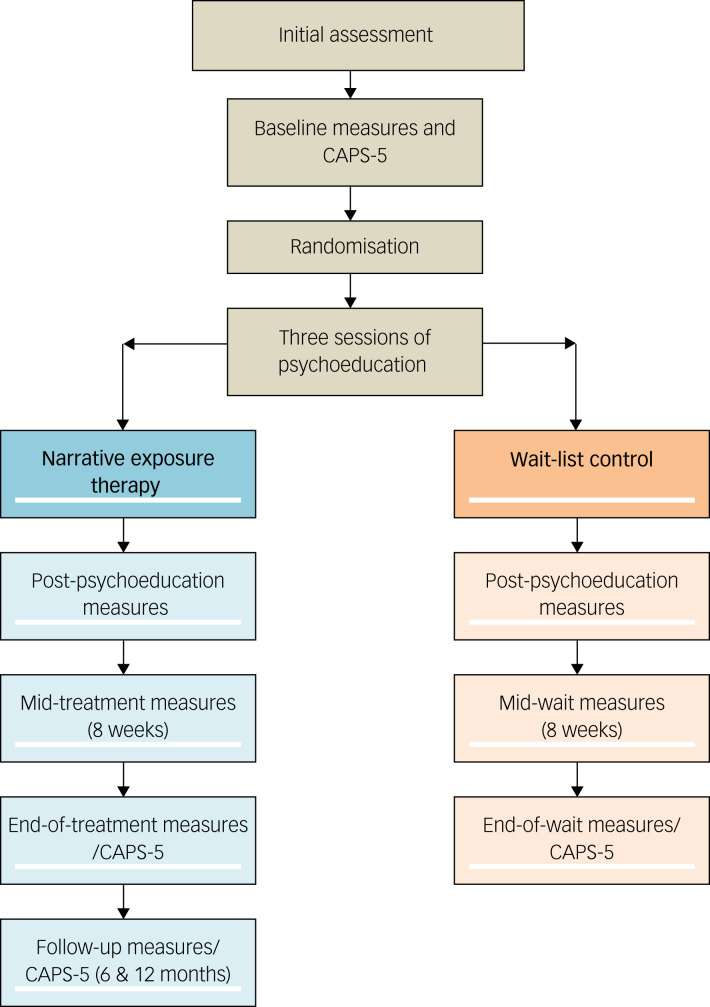
Study procedure.

This trial was designed to comply with the ethical standards of the relevant national and institutional committees on human experimentation (and with the Helsinki Declaration of 1975, as revised in 2008). The study was approved by the ethics committee of University College London (813/002). The procedure is summarised in Fig. 2.

In view of their past experiences of exploitation, ensuring participants could give informed (written) consent was crucial. Participants were provided with detailed verbal and written information, with professionally trained interpreter assistance where required. Multiple opportunities to withdraw consent were provided prior to commencing and during the study.

After diagnostic assessment and completion of baseline outcome measures, a research assistant randomised participants to either trial condition using a virtual coin toss programme. Clinical outcome measures were collected at baseline (pre-treatment), midway through treatment or the wait-list period, and at the end of treatment or the wait-list period. Standardised translations of the outcome measures were not consistently available in all languages required by participants. Interpreters were therefore used to complete standardised outcome measures where required. Participants randomised to the wait-list condition were offered NET after the study had concluded.

The aim was for all assessors working on the study to remain blind to allocation throughout the trial. Individuals allocated to the wait-list waited for 5 months before being reassessed and offered NET.

### Intervention

Irrespective of treatment condition, each participant was initially offered three sessions of protocolised psychoeducation about PTSD symptoms and symptom management strategies (broadly similar to that described in previous literature^23^). The standard NET protocol suggests only one session of psychoeducation.^8,9^ However, given the high clinical need of this population, we offered three sessions to increase the safety and acceptability of the waiting period for those randomised to the wait-list condition. Psychoeducation sessions were delivered by NET therapists and trained assistant psychologists. Interpreters were used where required. Participants then entered either the wait-list or the intervention phase.

NET sessions were delivered by seven female psychological therapists (six clinical psychologists and one psychotherapist); all had training and prior experience in working with survivors of trafficking and in delivering NET (trained by F.B., E.W. and K.R. and supervised by F.B. and E.W.). Treatment fidelity was monitored through the recording of therapy sessions and regular case supervision by clinical psychologists with extensive experience of NET. Sessions lasted 90 to 120 min and were offered on a weekly basis.

The first session of NET involved co-construction of a lifeline, in which the participant and the therapist construct a chronological overview of the events of the participant's life, including traumatic experiences.^8,9^ A therapy plan was then constructed collaboratively, taking into account the maximum number of 20 sessions available as dictated by the study protocol. This maximum number was agreed on by the authors in line with guidance provided by a clinician with expertise in NET (K.R.) and the available clinical resources. As directed by the NET manual, the majority of time in sessions was spent on facilitation of the participant's detailed narration of their traumatic experiences to ‘process’ the trauma memory, facilitate attachment repair, and make meaning of their traumatic and adverse experiences. Where time permitted, positive life events were also explored. A written narrative was created following the therapy sessions; this was read back and given to participants in the final therapy session.

Owing to the potential for instability or changes in the participants’ social and legal circumstances, a maximum of four additional non-NET sessions were also available for therapists to offer at their discretion to address any urgent or risk issues arising during treatment (see Results section).

### Feasibility

The study was intended to explore the feasibility of implementing a subsequent RCT. Our decision-making was informed by feasibility trial evaluation guidelines known as the ADePT Framework.^24^ Accordingly, we considered barriers to a large-scale trial, amendments needed to improve the success of a follow-on RCT, and the practicality of addressing these. These questions were considered in determining whether a follow-on RCT would be (a) feasible, (b) feasible with amendments or (c) not feasible.

### Data analysis

To investigate any change between pre-treatment and post-treatment across the intervention and the control group, we ran analyses of covariance (ANCOVA) for each outcome measure while controlling for baseline levels (in line with previous studies^25,26^).

## Results

### Feasibility

The ADePT framework^24^ was used to evaluate the feasibility outcomes of the study.

### Recruitment, sample size and retention

**Fig. 3:**
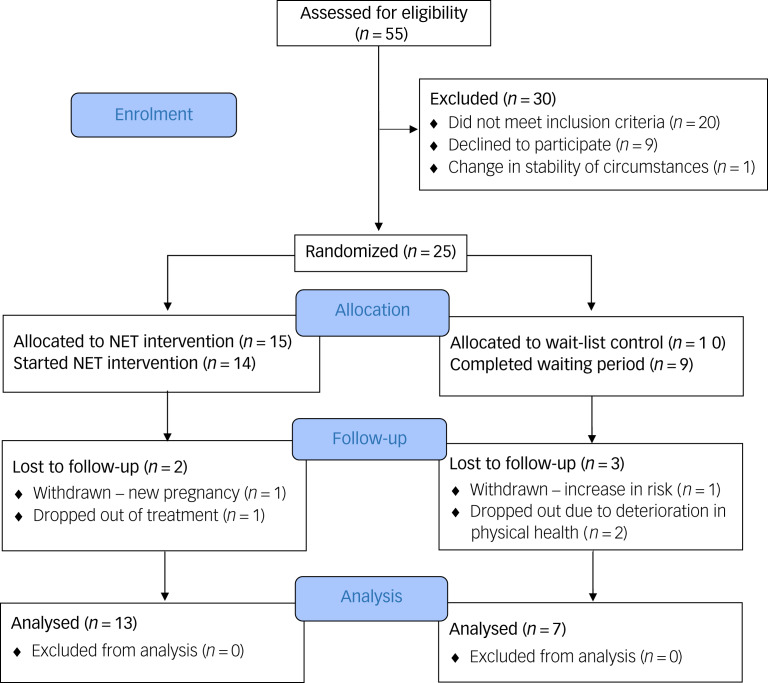
CONSORT participant flow diagram.

Details of participant flow and retention are shown in Fig. 3. Initially, we aimed to recruit 30 participants for the trial. Ultimately, 55 people were assessed, but 29 individuals did not meet the inclusion criteria for the following reasons: not having a diagnosis of PTSD (*n* = 10); not attending appointment(s) (*n* = 4); in crisis/high suicide risk (*n* = 2); previously received trauma-focused therapy (*n* = 2); preoccupation with instability in social or legal circumstances (*n* = 1); or still in exploitation (*n* = 1, necessary safeguarding procedures were followed). A further nine individuals met the inclusion criteria but declined to participate in the study for the following reasons: not wanting to engage in trauma-focused therapy at the time (*n* = 7); not willing to travel for appointments (*n* = 1); and not consenting to recording of therapy sessions (*n* = 1). Trial recruitment took longer than expected (approximately 16 months) and had to be concluded before the target number of 30 participants had been recruited. However, recruitment was sufficient overall to enable evaluation of feasibility.

Randomisation did not yield equal allocation across the two groups. There were also some notable group differences in the distribution of other participant characteristics, including whether the participant was an English speaker, and in terms of country of origin.

Overall, retention in both arms of the trial was acceptable. Within the NET group, one individual discovered that she was in the advanced stages of pregnancy soon after allocation. It was not feasible for her to receive a full course of treatment, so she was withdrawn from the study. One individual dropped out of therapy after eight sessions, stating that they no longer wanted to engage in trauma-focused therapy. All other participants in the NET group (*n* = 13) completed NET.

In the wait-list group, one participant was removed from the trial before the end of the waiting period owing to a significant increase in their risk of suicide. The remaining participants in the wait-list group completed the waiting period. However, two participants dropped out of the study at the point of post-wait assessment, owing to a deterioration in their physical health, and were therefore lost to follow-up. The remaining participants in the wait-list group were offered the opportunity to receive NET, and all took up this offer.

### Protocol adherence

Overall, the procedures set in place at the outset worked well during the study. Outlined below are the key areas of the protocol where significant challenges arose.

### Intervention

NET was generally perceived by participants to be an appropriate and acceptable treatment for their psychological difficulties. However, seven individuals declined to take part in the study because they did not feel ready to engage in a trauma-focused intervention at the point it was offered. One individual dropped out during therapy, citing the same reason. The reasons behind these decisions mostly related to feeling too unsettled in their social circumstances to focus on trauma therapy.

In all but one case, NET was delivered within the anticipated time frame of a maximum of 20 sessions (range 13–26 sessions). No substantial issues arose with regards to therapist adherence to the treatment model. Participants received a mean of 17 sessions of NET (s.d. = 4.07) across a mean of 220.6 days (s.d. = 65.27; approximately 7.4 months). This timeframe was longer than the 5–6 months anticipated. The main contributing factor to this was the varied regularity in session attendance, with a mean of 2.5 cancelled or non-attended sessions (range = 0–9) per participant over the course of therapy.

To ensure that we could fulfil the recruitment aims of the study, the trial included participants who were not able to access holistic support from the organisation to address other practical needs (owing to capacity limitations across the organisation). Many of these ‘trial-only’ participants had significant legal and social needs, but few had regular access to support agencies who could provide robust assistance with these issues.

Of those who completed NET, ‘trial-only’ participants (*n* = 6) received a mean of 2.67 (s.d. = 2.07) additional sessions to address other problems (such as newly arising legal and social problems, risk or mental health crises), compared with 1.71 (s.d. = 1.70) for those who had access to holistic support through the organisation (*n* = 7). The range was 0–5 across both groups. In the absence of other sources of support, therapists anecdotally reported needing to engage in other advocacy activities for participants, including preparing clinical letters in relation to immigration or housing issues. The importance of offering holistic support alongside psychological therapy is explored in more detail in the Discussion section.

### Assessments and data collection

The measures used appeared to be acceptable for participants and provided useful information about key clinical outcomes. Assessments of participants’ PTSD symptoms necessitated speaking about their trauma. Assessments were conducted by trained clinicians who could support participants if they became emotionally distressed. This procedure should be maintained for any future trial, given the extent of participants’ trauma histories.

Not all assessments were completed at all time points. Some participants dropped out because of a change in their circumstances. Some reported finding the length of assessment appointments and the number of assessments challenging. At times, participants did not prioritise attending these appointments, particularly in the follow-up period, once treatment was complete. Proactive engagement from the research team was required to encourage individuals to attend assessments. Despite these efforts, there were some delays or gaps in data collection, and some participants were lost to follow-up. No financial incentives were offered in this study. However, future studies should consider offering small financial or other incentives for participants to reimburse them for the time spent attending research assessments. This would recognise the vital contribution of the survivors to the research and hopefully improve data completeness.

### Adherence to blinding

The study research coordinator attempted to keep clinicians conducting assessments and outcome measures blind throughout the trial to minimise bias risk. Maintaining blinding was difficult within a small clinical team, and was a particular challenge because of the organisation's regular work activities, which sometimes resulted in a therapist having clinical contact with participants for non-trial reasons. Unblinding therefore occurred in several cases, either ‘accidentally’ (e.g. the assessor had encountered the participant attending for a therapy appointment) or because the participant made a disclosure during an assessment session, despite being asked not to do so. Larger-scale studies would benefit from having dedicated trial therapists or assessors to facilitate blinding adherence.

### Clinical outcomes

Given that this was a feasibility study with only a small sample and some missing data, we analysed completer data rather than performing an intention-to-treat (ITT) analysis. We would recommend that future full-scale RCTs are sufficiently powered that ITT analysis is possible.

**Fig. 4:**
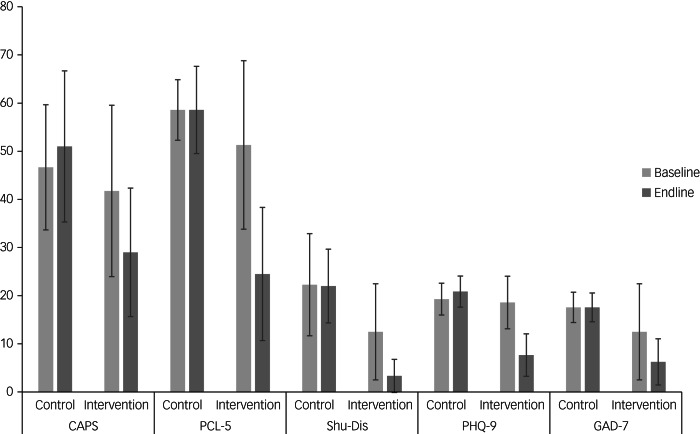
Scores for NET and wait-list groups at baseline and endline.

In terms of baseline scores, *t*-tests revealed no significant differences on the outcome measures across the two groups, except for the Shu-Dis (*t* = 2.19, *P* = 0.04), with those in the wait-list group reporting more dissociative symptoms (see Discussion section). A summary of the scores at baseline and endline (end of treatment or end of waiting period) can be seen in Fig. 4. Three data points were missing from the final analysis: two participants’ CAPS scores at baseline (one in the NET group, one in the wait-list group) and one endline PCL-5 score (NET group). Where data were missing, these participants were excluded from these specific analyses.

To investigate changes between baseline and endline across the NET and wait-list groups, we conducted ANCOVA for each outcome measure while controlling for baseline scores.

In line with our *a priori* hypothesis, there was a significant decrease in the scores of all outcome measures between pre- and post-treatment in the NET group (Table 2). However, in the wait-list group, no such change was observed and scores on most outcomes remained stable or increased slightly.

**Table 2:** ANCOVA results

	Coefficient	95% CI
NET *v*. wait-list	−18.42**	[−30.21, −6.62]
PCL-5 (*n* = 18)		
NET *v*. wait-list	−30.90***	[−42.16, −19.64]
Shu-Dis (*n* = 20)		
NET *v*. wait-list	−16.21***	[−21.76, −10.63]
PHQ-9 (*n* = 20)		
NET *v*. wait-list	−12.74***	[−16.60, −8.88]
GAD-7 (*n* = 20)		
NET *v*. wait-list	−10.35***	[−14.70, −6.00]

GAD-7, seven-item Generalised Anxiety Disorder Scale; PHQ-9, nine-item Patient Health Questionnaire.

**P < 0.01, ***P < 0.001.

No formal power calculation was completed for this feasibility study. However, the above results indicate a magnitude of change in scores on both the CAPS-5 and PCL-5 (the primary outcome variables) in the NET group that is statistically significant and reflects a large effect size (Cohen's *d* = 2.36).

In terms of clinically significant change in the NET group, 50% of individuals in the NET group (*n* = 12 owing to missing data) showed clinically significant change in their endline CAPS-5 scores. Of the remainder, two participants (16.6%) showed some worsening in scores, but this was not clinically significant. All other individuals showed a non-significant trend towards improvement.

Using the criteria outlined above, we also explored clinically significant change on the PCL-5 in the NET group. The results indicated that 83% of participants (*n* = 12 owing to missing data) showed clinically significant improvement in their self-reported PTSD symptoms at endline. Of the remaining two participants, one reported symptomatic improvement which was not clinically significant, and another reported a slight worsening of symptoms, although symptoms were only just above diagnostic threshold even at baseline.

## Discussion

Survivors of human trafficking experience multiple forms of abuse and suffer a range of health consequences.^2,3^ There is limited evidence relating to effective treatments for this complex and highly vulnerable group, and little is known about their ability to engage in psychological therapy.

This feasibility study shows that evidence-based psychological interventions can be implemented successfully with trafficked individuals with a chronic trauma history, even while they continue to experience uncertainty and instability in their immigration and social circumstances. The low attrition rate indicates that NET was perceived to be an acceptable treatment by trafficking survivors. The results are promising and suggest that NET is a viable treatment for survivors of trafficking that warrants further evaluation in full-scale RCTs.

However, a number of clinical and procedural issues would require consideration in any future trials involving trafficking survivors.

### Clinical issues

Although participant recruitment and retention were acceptable, a number of individuals were not sufficiently stable or declined to participate in a trauma-focused intervention. This is in keeping with the emergent literature on the provision of psychosocial care to survivors of trafficking, which suggests that their other (often significant) needs, including safety, social and legal needs, must be addressed alongside any mental health treatment.^27^ Comprehensive case management to address the full range of psychosocial needs of the survivor is important to support positive clinical outcomes. However, many participants completed (and benefited from) NET despite their ongoing practical problems. This suggests that ongoing legal and social problems are not necessarily a barrier to an individual's engagement with a trauma-focused intervention.

A collaborative approach, with respect for survivors’ autonomy, is considered to be another pillar of clinical interventions for trafficking survivors. Providing detailed information about treatment and supporting survivors to make an informed choice about their care is particularly important for this population. A key challenge in delivering an RCT with this population was the lack of flexibility in how and when treatment could be offered, which is known to be an important consideration when working clinically with this group.^4,6^ Although uptake of and engagement with the intervention within the constraints of the RCT were generally good, further consideration should be given to how the RCT protocol might be adapted to include more flexibility for individuals who are also dealing with substantial social problems, for example, offering a staggered baseline design.

Although NET brought about a reduction in both PTSD and other psychological symptoms, some clinical issues emerged that require further exploration in future research. The participants were quite heterogenous as a group: some reported a long trauma history that extended beyond their trafficking experiences, whereas others had fewer or more time-limited exposures to trauma. This led to high variability in baseline and outcome scores and made it difficult to draw conclusions that would be fully generalisable to survivors of all types (or lengths) of trafficking. It also necessitated decisions about which traumatic events could be addressed within a limited number of sessions. This was sometimes challenging both for participants and therapists. Trafficking often leaves individuals with significant trust difficulties, but the limitations of the trial necessitated therapists to quickly develop a trusting working alliance with the participants. As above, consideration should be given to where it might be possible to introduce flexibility into the treatment protocol to optimally facilitate survivor engagement in trauma-focused treatment.

In addition, the impact of shame in the maintenance of PTSD symptoms (and as a barrier to engagement with trauma-focused treatment) frequently arose as a theme in therapy sessions and in the therapists’ supervision sessions. This is in keeping with previous research,^10,13^ which also highlights shame as a key difficulty for trafficking survivors. This issue may benefit from further exploration in future research, for example, ascertaining whether any particular adaptations to the standard NET protocol might be helpful for this group. However, of note is that the final outcomes were post-treatment, whereas research^28^ indicates benefits of NET over longer follow-up periods.

Further research should include a follow-up period and could also explore the experience of trafficking survivors undertaking NET and factors that influence decision-making around engaging in treatment. Consideration should also be given to developing and trialling alternative interventions (drawing on the existing evidence base) that might help meet the needs of trafficking survivors with symptoms of PTSD who do not yet feel ready to engage with trauma-focused therapy.

### Impact of practical needs of trafficking survivors

Owing to the study's relatively flexible inclusion criteria, only two individuals were excluded from the trial on the grounds of preoccupation with social or legal issues. The majority of participants had unresolved immigration claims at the point of entry to the trial. We had anticipated delivering treatment in the absence of integrated holistic support, but inconsistency in participants’ access to practical support (including legal protection) was a key challenge in this study. For individuals who did not have access to the organisation's holistic model of care, the therapist was often the sole provider of care and undertook additional advocacy activities to ensure the participant's safety and stability. At times, this advocacy work was also required to manage wavering engagement in NET due to preoccupation with legal and welfare issues.

Although we had anticipated that trafficking survivors would have additional practical needs, we had expected that ‘trial-only’ participants would be able to access alternative sources of practical support in the community during the course of therapy (such as via the agency that referred them into the trial). However, very few ‘trial-only’ participants were able to access adequate practical support, and they relied heavily on their trial therapist, which had not been expected at the design stage of the study. The sample size was too small to make meaningful comparisons in relation to this (although the number of trial-only participants was distributed relatively evenly across the NET and wait-list groups). During the implementation of the trial, we decided that it was clinically unethical not to provide equal access to support for urgent needs (e.g. for legal or housing issues); the trial therapists therefore spent time not recorded in the session total ensuring these needs were met (e.g. through writing clinical letters). We also did not record the amount of contact that the organisational clients may have had with other team members in order to address any practical needs. This is a limitation and may have affected our clinical outcomes because some individuals may have accessed more support than others during the treatment or wait-list period.

We have reflected on how future studies might address this issue. The main challenge in manualising access to practical support is the unpredictability of needs. Even individuals with leave to remain in the UK and relatively stable circumstances at trial commencement were not exempt from experiencing significant unpredictable problems (e.g. housing or financial crises) over the course of the project. We suggest that to strike a balance between methodological rigour and clinically ethical practice, it is important that *ad hoc* practical support is available to all participants, rather than participants being offered a manualised ‘dose’ of practical support sessions. It will also be crucial to monitor how and when such support is utilised by participants in a future study to explore what (if any) relationship this has with treatment outcomes.

There is an ethical question about the appropriateness of a wait-list control for trafficking survivors in unstable psychosocial circumstances. Two participants in the wait-list group were too unstable to take up therapy when it was later offered owing to deterioration in their circumstances. This issue should be considered carefully both in future RCTs and in clinical settings, where it might be prudent to prioritise survivors of trafficking on therapy waiting lists so that therapy is offered at a time when the individual is in a period of relative stability. At a minimum, it is important that survivors have ready access to *ad hoc* holistic support throughout any waiting period, which might reduce the risk of people destabilising or dropping out before therapy can be offered.

A key clinical recommendation of this study is that psychological support for trafficking survivors should be provided within the context of multidisciplinary support. This is likely to promote sustained engagement with psychological treatment and consequently more optimal outcomes from therapy. This is echoed in literature^4,13,27^ which notes that beyond psychological treatment, there is a need for trafficking survivors to access adjunctive support, to promote safety and stability and reduce the risk of future exploitation. Future RCTs should therefore make provision for multidisciplinary support to flexibly address the holistic needs of participants within the trial context.

### Procedural issues

The clinical setting of the trial led to some challenges with delivering the RCT protocol and assessments exactly as planned. One issue was that randomisation did not bring about an even distribution of key demographic characteristics of the participants across the groups, which could have influenced the results. Of particular note, a significant difference in scores on the Shu-Dis (self-rated dissociation symptoms) was found between the groups at baseline. This may have influenced the results of the study and may be relevant to treatment outcomes. Future studies should therefore consider using stratification to ensure better matching between groups, including on factors such as level of dissociative symptoms. In addition, although the clinical findings of this study are promising, the small sample size may have inflated the magnitude of the effect size, so further studies with a larger sample are required. Similarly, the immediate post-treatment improvement for NET clients may have been a placebo effect, and post-treatment follow up of at least three months should be included in future trials.

Another threat to the validity of the treatment outcomes was the use of interpreters to translate outcome measures in the study. Although this was necessary to avoid excluding non-English speakers from the study, the lack of availability of standardised translations of these measures may have led to bias in translation or understanding of measures. This may have affected the overall results of the study. Future trials should endeavour to use standardised translations of outcome measures wherever possible (or to translate and back-translate measures).

Owing to limited resources, there were a high number of therapists involved in the delivery of NET and trial assessments in this study. This may have led to some inconsistencies across treatment and assessments. Treatment model fidelity was closely monitored through supervision, but the lack of an objective structured fidelity rating was a limitation of this study.

In addition, some individuals received more than the allocated number of NET (or non-NET) sessions, because clinicians used their discretion to extend the course of therapy where this was clinically indicated, where participants missed or cancelled sessions, or where additional non-therapy sessions were required to address practical issues. This led to an extended average period of time over which therapy was delivered (over 7 months), resulting in a discrepancy with the control wait-list period, which was maintained at 5 months (the original estimated duration of therapy). This may have influenced the results of the study to some degree. The NET session number protocol should be monitored and adhered to more closely in any full-scale trial, which should be more possible if practical support is available consistently to all participants. Consideration should also be given to whether more flexibility is needed in the number of sessions offered for individuals with very chronic trauma histories. To address these procedural issues, it is recommended that dedicated trial clinicians are identified to undertake any assessment or therapy delivery within future RCTs.

In conclusion, the results of this study indicate that a full-scale RCT is likely to be feasible with trafficking survivors in a real-world clinical context. The findings build on the existing literature indicating that NET is an effective treatment for survivors of trafficking presenting with PTSD and can bring about improvement in other psychological symptoms. Future research should consider ways to ensure the holistic needs of participants are met, to facilitate their optimal engagement in any psychological intervention.

## Data Availability

The data that support the findings of this study are available on request from the corresponding author (F.B.).
